# On the Role of Cs_4_PbBr_6_ Phase in the Luminescence Performance of Bright CsPbBr_3_ Nanocrystals

**DOI:** 10.3390/nano11081935

**Published:** 2021-07-27

**Authors:** Kateřina Děcká, Adéla Suchá, Jan Král, Ivo Jakubec, Martin Nikl, Vítězslav Jarý, Vladimir Babin, Eva Mihóková, Václav Čuba

**Affiliations:** 1 Department of Nuclear Chemistry, Faculty of Nuclear Sciences and Physical Engineering, Czech Technical University in Prague, Břehová 7, 115 19 Prague, Czech Republic; suchaade@fjfi.cvut.cz (A.S.); kralja13@fjfi.cvut.cz (J.K.); vaclav.cuba@fjfi.cvut.cz (V.Č.); 2 Institute of Physics, Czech Academy of Sciences, Cukrovarnická 10, 162 00 Prague, Czech Republic; nikl@fzu.cz (M.N.); jary@fzu.cz (V.J.); babinv@fzu.cz (V.B.); mihokova@fzu.cz (E.M.); 3 Institute of Inorganic Chemistry, Czech Academy of Sciences, Husinec-Řež č.p. 1001, 250 68 Řež, Czech Republic; jakubec@iic.cas.cz

**Keywords:** nanocrystals, lead halide perovskites, luminescence, scintillation detectors

## Abstract

CsPbBr_3_ nanocrystals have been identified as a highly promising material for various optoelectronic applications. However, they tend to coexist with Cs_4_PbBr_6_ phase when the reaction conditions are not controlled carefully. It is therefore imperative to understand how the presence of this phase affects the luminescence performance of CsPbBr_3_ nanocrystals_._ We synthesized a mixed CsPbBr_3_-Cs_4_PbBr_6_ sample, and compared its photo- and radioluminescence properties, including timing characteristics, to the performance of pure CsPbBr_3_ nanocrystals. The possibility of energy transfer between the two phases was also explored. We demonstrated that the presence of Cs_4_PbBr_6_ causes significant drop in radioluminescence intensity of CsPbBr_3_ nanocrystals, which can limit possible future applications of Cs_4_PbBr_6_-CsPbBr_3_ mixtures or composites as scintillation detectors.

## 1. Introduction

Cesium lead halide perovskite quantum dots of the CsPbX_3_ (X = Cl, Br, I) formula have been first identified by Nikl et al. group as nanoinclusions in CsX host doped by Pb^2+^ ions [1,2,3]. However, they have not been studied extensively since the introduction of their colloidal synthesis in 2015 [4]. They were immediately identified as highly promising materials for various applications, mostly for solar cells [5], LEDs [6], or displays [7]. Their excellent luminescent properties, such as high quantum efficiency, narrow emission lines, and fast decay times, are also highly desirable for scintillator manufacture. Recently, a body of studies on the lead halide perovskites has also been focused on their application in X-ray detection [8,9,10,11,12,13,14].

Nevertheless, this material also has some drawbacks; in particular its poor chemical stability on air [15,16]. An obvious solution would be provided by encapsulation of CsPbX_3_ in various inert matrices such as SiO_2_ [17,18,19], TiO_2_ [20], or organic polymers [8,21,22]. Many studies also proposed an interesting composite material CsPbBr_3_@Cs_4_PbBr_6_ which, besides enhanced stability, also passivates CsPbBr_3_ nanocrystals, i.e., suppresses non-radiative recombinations on surface defects [23,24,25]. Various CsPbBr_3_-Cs_4_PbBr_6_ mixtures in the form of two different nanocrystal population were also prepared [26,27].

Cs_4_PbBr_6_ is a material often referred to as a “zero-dimensional perovskite”, while CsPbBr_3_ is called a “three-dimensional perovskite”. CsPbBr_3_ consists of corner sharing PbBr_6_^4−^ octahedra, whereas in Cs_4_PbBr_6_ those octahedra are isolated (see Figure S1 in Supplementary Information). Cs_4_PbBr_6_ continues to be somewhat controversial material; there is still an ongoing debate on whether or not it is a source of bright green luminescence and, if so, what the origin of that luminescence is [28]. There is also a question how the presence of Cs_4_PbBr_6_ affects the luminescent properties of CsPbBr_3_ and vice versa.

The debate was initiated by some early reports on pure Cs_4_PbBr_6_ crystals with superior green luminescence without any profound considerations about the origin of such luminescence [29,30]. The origin of the green luminescence was questioned, and two major opinions appeared in research community; one strong opinion is that CsPbBr_3_ nanoinclusions are, in fact, present in Cs_4_PbBr_6_ crystals [25,31,32,33], as the bright green emission is associated with CsPbBr_3_ nanocrystals. It has already been stated in 1999 by Nikl et al. that it is difficult to suppress the presence of CsPbBr_3_ in Cs_4_PbBr_6_ completely [34]. This point of view is further supported by many studies on non-luminescent Cs_4_PbBr_6_ that can be easily transformed into bright CsPbBr_3_ [35,36,37,38,39].

The other strong opinion proposes that the green luminescence is due to point defects in Cs_4_PbBr_6_ structure. Some possible defects that may cause green luminescence were identified, for example the Br vacancy [40,41,42]. For more details, please refer to review papers published on this subject, for example the most recent, [28], which supports the opinion based on the presence of nanoinclusions, and provides persuasive arguments rebutting the Br vacancy concept.

Understanding of the role of CsPbBr_3_ and Cs_4_PbBr_6_ phases in the luminescence of cesium lead bromides is particularly important when considering applications and future needs to scale-up the production for manufacturing. CsPbBr_3_ nanocrystals have been recently identified as highly prospective scintillators for applications requiring fast response, for example a new generation of time-of-flight positron emission tomographs (TOF-PET), or new detectors for high energy physics [43,44]. However, these considerations are important regardless the target application. It is clear that CsPbBr_3_ and Cs_4_PbBr_6_ phases tend to coexist. Therefore, it is evident that this tendency may become a serious issue in a scale-up of the synthesis for industrial production. In order to manufacture a material of the best performance, it is imperative to know how detrimental a contamination of CsPbBr_3_ nanocrystals by Cs_4_PbBr_6_ phase can be, if at all. There have already been some arguments raised in the recent literature against the possible applicability of CsPbBr_3_@Cs_4_PbBr_6_ composite as a scintillator [45].

The band gap energy of Cs_4_PbBr_6_ and CsPbBr_3_ was calculated to be 3.9 eV and 2.3 eV, respectively [46]. This allows an energy transfer from Cs_4_PbBr_6_ to CsPbBr_3_. This transfer can be both radiative and/or non-radiative. Excitation in Cs_4_PbBr_6_ phase results in formation of self-trapped excitons that radiatively recombine while emitting UV photons. This emission can radiatively excite CsPbBr_3_. The band alignment in the core-shell structure CsPbBr_3_@Cs_4_PbBr_6_ is of the type I, which means that the valence band maximum and the conduction band minimum are fully within the Cs_4_PbBr_6_ band gap. This also allows a non-radiative energy transfer from Cs_4_PbBr_6_ to CsPbBr_3_ by hopping [46].

However, when the energy transfer does not occur, the presence of Cs_4_PbBr_6_ may hinder the luminescence from CsPbBr_3_. In a theoretical model of 80 nm slab of CsPbBr_3_ below 10 μm of CsPbBr_3_@Cs_4_PbBr_6_ composite, the escaping emission spectrum was calculated to be 100× attenuated compared to the launched spectrum from the CsPbBr_3_ slab [38]. The attenuation coefficient of Cs_4_PbBr_6_ is higher than that of CsPbBr_3_ [47], therefore the incident energy will be preferably deposited in the Cs_4_PbBr_6_ phase.

This study intends to contribute to an intense and important debate about the CsPbBr_3_ vs. Cs_4_PbBr_6_ issue, and also to shed some light on the (radio)luminescence properties of CsPbBr_3_ and Cs_4_PbBr_6_ mixtures, which should help to better understand the dynamics of the abovementioned CsPbBr_3_@Cs_4_PbBr_6_ composite and its applicability in the field of scintillation detectors. In particular, analysis of radioluminescence decays of our materials might provide a valuable set of data on the light and/or energy transfer between the two phases. We reiterate that, unlike rather extended literature on PL properties of materials in question, data on scintillation properties, especially scintillation decays, are scarce.

We synthesized CsPbBr_3_ nanocrystals using the hot injection method (HI) [4] and their mixture with Cs_4_PbBr_6_ crystals using the room-temperature precipitation method (RTP) [48]. The RTP method is, by its nature (simple mixing of two solutions without any heating), the best candidate for potential scaling up. The HI is the most widely used method, which proves its robustness and reproducibility. We found out that the HI method usually leads to high quality pure CsPbBr_3_ nanocrystals, while RTP protocol resulted in various CsPbBr_3_-Cs_4_PbBr_6_ mixtures. We studied and compared luminescent properties of all samples in detail (both photoluminescence and radioluminescence, including decay kinetics) with respect to their composition, structure, and morphology. We found out that the presence of Cs_4_PbBr_6_ phase significantly deteriorates CsPbBr_3_ scintillation light output, which can limit the application potential of CsPbBr_3_-Cs_4_PbBr_6_ mixtures as scintillation detectors.

## 2. Materials and Methods

### 2.1. Chemicals

This study utilizes the following chemicals: CsBr (99.999%, Merck, Darmstadt, Germany), PbBr_2_ (99.999%, Merck, Darmstadt, Germany), Cs_2_CO_3_ (99.9%, Merck, Darmstadt, Germany), oleylamine (OAm, 70%, Merck, Darmstadt, Germany), oleic acid (OA, 90%, Merck, Darmstadt, Germany), 1-octadecene (90%, Merck, Darmstadt, Germany), n-hexane (anhydrous, 98%, Merck, Darmstadt, Germany), toluene (99.8%, Merck, Darmstadt, Germany), and *N,N*–dimethylformamide (DMF, anhydrous, 99.8%, Merck, Darmstadt, Germany). All chemicals were used as received, without further purification, unless stated otherwise.

### 2.2. Hot Injection (HI) Synthesis of Pure CsPbBr_3_

The procedure introduced by Protesescu et al. was used [4]. In short, 0.752 mmol of PbBr_2_, 20 mL of 1-octadecene (ODE), 2 mL of oleylamine (OAm), and 1.78 mL of oleic acid (OA), were mixed in 100 mL 3-necked flask and degassed at 110 °C under vacuum for 1 h. After that, 0.5 mL of dried pre-synthesized cesium oleate solution (0.4 M) was injected at 170 °C under argon atmosphere. Solid product was separated from ODE solution by centrifugation and redispersed in hexane. For narrowing the size distribution and enhancing colloidal stability, one more centrifugation step was preformed, and the supernatant was collected. 

The synthesis of cesium oleate was modified according to the study by Lu [49], which provides a complete conversion of cesium salt to cesium oleate, resulting in better reproducibility of synthesis, and in complete solubility of cesium oleate at room temperature by reacting 5 molar equivalents of oleic acid with respect to Cs. The amount of OA added during the CsPbBr_3_ synthesis was adjusted to match the molar ratios from [4]. 

For more details on both syntheses, please refer to Supplementary Information.

### 2.3. Room-Temperature Precipitation (RTP) Synthesis of CsPbBr_3_-Cs_4_PbBr_6_ Mixture

The procedure introduced by Li et al. [48] as supersaturation recrystallization (currently called room-temperature precipitation) was used with slight modifications for better reproducibility. In short, 0.4 mmol of PbBr_2_ and 0.4 mmol of CsBr were dissolved in 10 mL of dimethylformamide (DMF) and 1 mL of OA and 0.5 mL of OAm were added. Then, 1 mL of the solution was quickly added to 10 mL of toluene. Solid product was collected by centrifugation, both the supernatant and the precipitate were characterized. For more details, please refer to Supplementary Information.

### 2.4. Characterization

X-ray powder diffraction (XRPD) was measured using a Rigaku Miniflex 600 diffractometer equipped with the Cu X-ray tube (average wavelength K_α1,2_ 0.15418 nm, voltage 40 kV, current 15 mA). Data were collected with a speed of 2°/min and compared with the ICDD PDF-2 database, version 2013. The transmission electron microscopy (TEM) was obtained using an EM201 microscope (Philips, Amsterdam, Netherlands). Absorption spectra were collected using a Cary 100 spectrophotometer (Varian, Palo Alto, CA, USA). Photoluminescence (PL) excitation and emission spectra were collected using a FluoroMax spectrofluorometer (Horiba Jobin Yvon, Kyoto, Japan). Radioluminescence (RL) spectra were collected using a 5000 M spectrofluorometer (Horiba Jobin Yvon, Kyoto, Japan) with a monochromator and TBX-04 (IBH Scotland, Glasgow, Scotland) photodetector, the excitation source was a Seifert X-ray tube (40 kV, 15 mA). Spectrofluorometer 5000 M (Horiba Jobin Yvon, Kyoto, Japan) was used for measuring PL decay curves using the pulsed nanoLED sources (IBH Scotland, Glasgow, Scotland, excitation wavelengths 310 nm and 389 nm, 80 kHz repetition rate) as the excitation sources. The detection part of the setup involved a single-grating monochromator and a photon counting detector TBX-04 (IBH Scotland, Glasgow, Scotland). RL decay curves were collected using hybrid picosecond photon detector HPPD-860 and Fluorohub unit (Horiba Scientific, Kyoto, Japan). Decays were recorded in both the long and short time windows, as the short time window is relevant for the fast timing applications. Samples were excited by picosecond (ps) X-ray tube N5084 from Hamamatsu, operating at 40 kV. The X-ray tube was driven by the ps light pulser (Hamamatsu, Hamamatsu City, Japan) equipped with a laser diode operating at 405 nm. The instrumental response function FWHM of the setup is about 76 ps. Convolution procedure was applied to all decay curves to determine true decay times (SpectraSolve software package, Ames Photonics, Hurst, TX, USA). The contribution of a component expressed as a percentage (often referred to as a light sum, LS) was calculated as:

$${\mathrm{LS}}_{n}=\;\frac{A_{n}\tau_{n}}{\sum A_{i}\tau_{i}}$$

where $A_{n}$ and $\tau_{n}$ denotes amplitude and decay time of the nth component.

XRPD, RL spectra, and RL/PL decays were measured on solid samples, i.e., precipitates after the first centrifugation step. In case of supernatant of sample prepared by RTP, XRPD was measured on drop-casted film. Samples for TEM characterization were obtained by drop-casting the final toluene/hexane solutions on TEM grid. Absorption and PL excitation/emission spectra were also collected on final toluene/hexane solutions.

## 3. Results and Discussion

**Figure 1:**
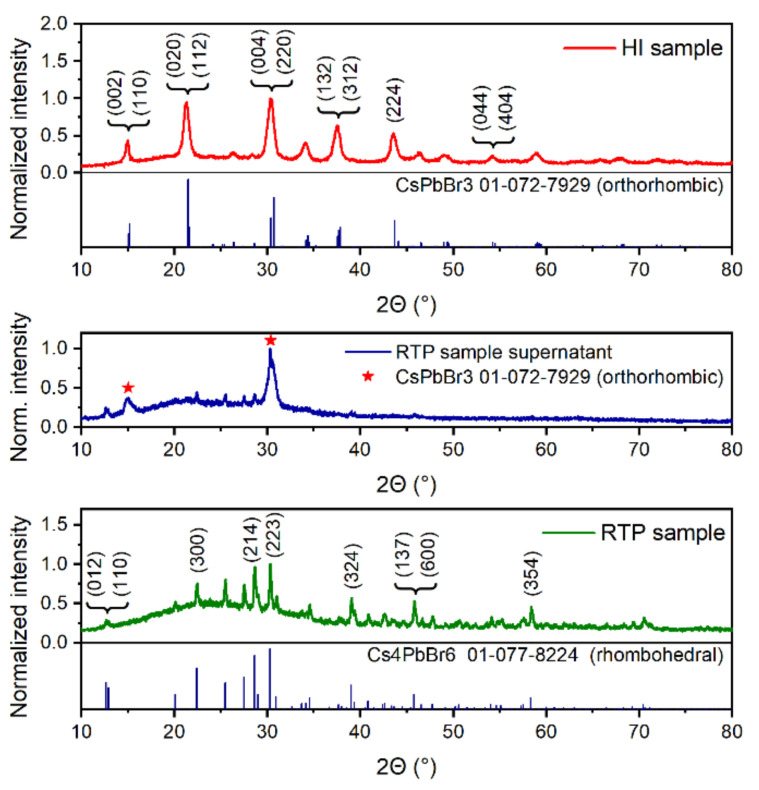
(From top to bottom) XRPD pattern of a precipitate of the sample prepared by the hot injection method (HI, red line) compared to the ICDD PDF-2 database record for CsPbBr_3_; XRPD pattern of a supernatant of the sample prepared by the room-temperature precipitation method (RTP, blue line), red stars denote positions of the most intense CsPbBr_3_ diffraction double lines; and XRPD pattern of a precipitate of the sample prepared by the RTP method (green line) compared to the ICDD PDF-2 database record for Cs_4_PbBr_6_.

XRPD patterns of all samples are presented in Figure 1 and compared to ICDD PDF-2 database records for orthorhombic CsPbBr_3_ (#01-072-7929) and rhombohedral Cs_4_PbBr_6_ (#01-077-8224) phases. The sample prepared by the hot injection (HI) method (red line) was identified as pure CsPbBr_3_ sample. The diffraction lines are significantly broadened, suggesting that this phase consists of very small crystallites. Halder–Wagner method of determining linear size of crystallites (using Scherrer constant value 0.94) revealed their mean size as (13 ± 1) nm. A slightly elevated background under 40°, suggesting the presence of an amorphous phase, can be attributed to a small excess of organic ligands (oleic acid and oleylamine) present in the measured sample.

Two diffractograms were recorded for the sample prepared by the room-temperature precipitation method (RTP); precipitated solid sample (green line in Figure 1) and supernatant from centrifugation (blue line). The pattern of the precipitate shows only the presence of Cs_4_PbBr_6_ phase and elevated background under 40° (i.e., an amorphous phase is present). A higher amount of an amorphous phase suggests a large excess of free organic ligands in this sample. Narrow peaks indicate that this phase has much larger crystallites than those of CsPbBr_3_ phase identified in the pure (HI) sample.

To prove the expected presence of CsPbBr_3_ nanocrystals in this sample (which was strongly indicated by blue/green luminescence of the sample, see below and also [3,25,31,47]), we also measured a drop-casted film of this sample’s supernatant (blue line in Figure 1). Narrow peaks of much lower intensity than in centrifuged sample remain present in this diffractogram. In addition, two broad peaks are present at around 15° and 30° (indicated by red stars). Detailed analysis revealed that the first peak can be attributed to two CsPbBr_3_ diffractions from (002) and (110) lattice planes, and the second peak can be attributed to CsPbBr_3_ diffractions from (004) and (220) lattice planes. This clearly indicates preferential orientation of nanocrystalline phase in this direction, suggesting the presence of nanoplatelets. As we have demonstrated, this type of synthesis is indeed capable of producing CsPbBr_3_ nanoplatelets in the supernatant [50]. Nevertheless, Figure 1 still provides only a partial evidence of the CsPbBr_3_ nanocrystals present in the centrifuged sample, as there are many CsPbBr_3_ diffraction lines missing in the pattern.

**Figure 2:**
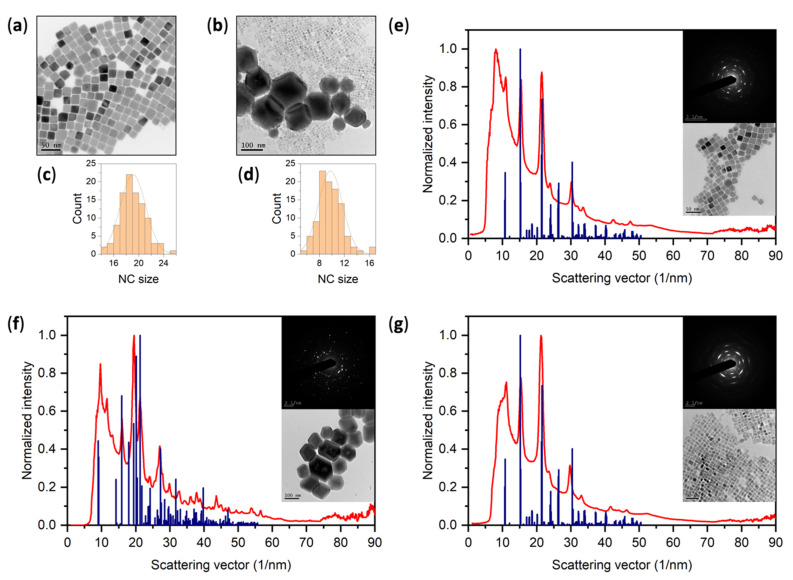
(**a**) TEM micrograph of the pure sample; (**b**) TEM micrograph of the mixed sample; (**c**) size distribution of 100 crystals presented in (**a**), the mean size is (19.1 ± 0.2) nm; (**d**) size distribution of 100 crystals presented in (**b**), the mean size is (9.8 ± 0.2) nm; (**e**) integrated radial intensity profile from a SAED pattern (in the inset) of the pure sample (corresponding micrograph in the inset) compared to the ICDD PDF-2 record for CsPbBr_3_ #01-072-7929; (**f**) integrated radial intensity profile from a SAED pattern (in the inset) of large hexagonal crystals present in the mixed sample (corresponding micrograph in the inset) compared to the ICDD PDF-2 record for Cs_4_PbBr_6_ #01-077-8224; and (**g**) integrated radial intensity profile from a SAED pattern (in the inset) of small cubic crystals present in the mixed sample (corresponding micrograph in the inset) compared to the ICDD PDF-2 record for CsPbBr_3_ #01-072-7929. Sizes of nanocrystals were measured using an ImageJ software [51] and SAED patterns were integrated using the ProcessDiffraction software [52].

To provide a stronger evidence of the CsPbBr_3_ presence, we performed TEM and analyzed selected area electron diffraction (SAED) patterns of the corresponding micrographs (see Figure 2). TEM in Figure 2a shows that the sample prepared by the HI method (identified by XRPD in Figure 1 as a pure orthorhombic CsPbBr_3_ sample with nano-sized crystallites), indeed consists of nanocrystals of cubic shape with the mean size of (19.1 ± 0.2) nm. This value is in good agreement with the calculated mean crystallite size from XRPD pattern in Figure 1. The small discrepancy may be caused by an inaccuracy of determining the FWHM (full width at half maxima) of CsPbBr_3_ orthorhombic double peaks and the fact that diffractions at larger angles are partially hidden in the background. TEM in Figure 2b shows that the sample prepared by the RTP method (identified as rhombohedral Cs_4_PbBr_6_ by XRPD in Figure 1), is clearly a mixture of larger hexagonal crystals (crystal size around 110 nm), and small nanocrystals of roughly cubic shape with the mean size of (9.8 ± 0.2) nm. SAED analysis in Figure 2e–g shows that, in both cases, the cubic nanocrystals can be attributed to the CsPbBr_3_ phase, while the hexagonal phase was confirmed as that of Cs_4_PbBr_6_. We conclude that the sample prepared by RTP method is, in fact, a mixed sample containing both the CsPbBr_3_ nanocrystals and the larger Cs_4_PbBr_6_ crystals.

The reason we cannot see the CsPbBr_3_ phase on XRPD clearly (only partially in the supernatant sample) is that Cs_4_PbBr_6_ crystals are one order of magnitude larger than CsPbBr_3_ nanocrystals. In this case, XRPD is not capable of distinguishing the CsPbBr_3_ phase present in minority, especially when consisting of smaller particles. We estimate that the amount of CsPbBr_3_ phase in this sample was less than 5%. Any reflections from CsPbBr_3_ nanocrystals are in this case destined to be lost in the background. CsPbBr_3_ reflections were observable only on the supernatant sample, as the majority of large Cs_4_PbBr_6_ crystals were separated by centrifugation. However, due to the preferential orientation, which resulted from the drop-casting process, this XRPD analysis was not conclusive enough. When in any doubt, it is crucial to exploit more sensitive methods, such as SAED, which was performed in this work, or for example using the synchrotron radiation for XRPD analysis, to avoid any misleading preliminary conclusions.

Based on the XRPD analysis, we denote the HI-prepared sample as “the pure sample” and the RTP-prepared sample as “the mixed sample”.

**Figure 3:**
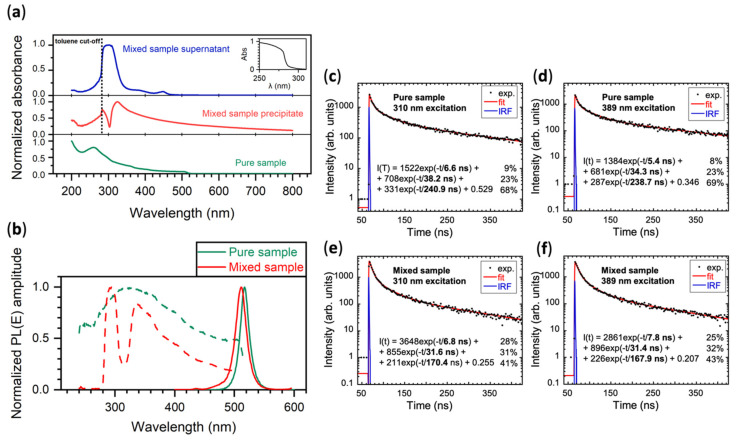
(**a**) Absorption spectra of both the supernatant (blue line) and precipitate (red line) of the mixed sample (in toluene) and that of the pure sample (green line, in hexane), in the inset: absorption spectrum of toluene; (**b**) PL characteristics of the pure sample (green lines) and the mixed sample (red lines), excitation spectra, collected at the emission maxima, are in dashed lines, emission spectra in solid lines, excitation wavelength was 300 nm in both cases; and (**c**–**f**) PL decay curves of both the pure (**c**,**d**) and mixed (**e**,**f**) samples, excited at 310 nm (**c**,**e**) and 389 nm (**d**,**f**). Black dots represent the experimental data, red line is the best fit (3-exponential function), and blue line is the instrumental response function (IRF).

Absorption spectra of all samples are presented in Figure 3a. Spectrum of pure CsPbBr_3_ sample (green line) features typical CsPbBr_3_ absorption edge at 515 nm. Absorption band peaking at 261 nm can be attributed to an excess of surfactants (this peak tends to diminish with lower concentration of nanocrystals, see the Supplementary Information Figure S2 for detailed explanation and additional spectra).

Absorption spectrum of the mixed sample (red line) has a very high background caused by the light scattering at large Cs_4_PbBr_6_ crystals. We can identify broader absorption band peaking between 305–330 nm, which may be attributed to the bulk absorption of Cs_4_PbBr_6_ crystals [10,34,53]. Any possible CsPbBr_3_-related absorption edge is hidden in the background. The spectrum rapidly drops down below 283 nm; this is caused by the toluene cut-off (see the toluene absorption spectrum in the inset). The same artefact can be observed in the absorption spectrum of the supernatant of the mixed sample, but not in the spectrum of the pure sample, which is dispersed in hexane.

Absorption spectrum of the supernatant of the mixed sample is presented as a blue line in Figure 3a. This spectrum features two CsPbBr_3_-related absorption maxima at 449 nm and 385 nm, both significantly blue-shifted compared to the absorption of the pure sample. As discussed above (XRPD characterization), CsPbBr_3_ nanocrystals present in this sample are probably in the form of nanoplatelets. Strong blue shift of absorption spectrum indicates that at least one dimension is below the exciton Bohr diameter (~4–7 nm) [4,50], which further supports the nanoplatelets hypothesis. Based on this consideration, we may attribute the 385 nm and 449 nm absorption features to the light hole-electron and heavy hole-electron transitions, respectively. Another feature in this spectrum is an absorption at 310 nm, which can be attributed to absorption of Cs_4_PbBr_6_ crystals [34,54].

Figure 3b shows photoluminescence (PL) emission and excitation spectra of both samples. Spectra of the mixed sample’s supernatant are presented in Supplementary Information Figures S3–S5. Emission maximum of the mixed sample is blue shifted from the maximum of the pure sample by 6 nm, which is caused by the size difference of CsPbBr_3_ nanocrystals. Excitation spectrum of the pure sample follows its absorption spectrum up to its maximum at 329 nm. However, excitation spectrum of the mixed sample features a significant drop in its intensity at 314 nm, which matches the Cs_4_PbBr_6_ absorption (similarly as in [55]).

**Figure 4:**
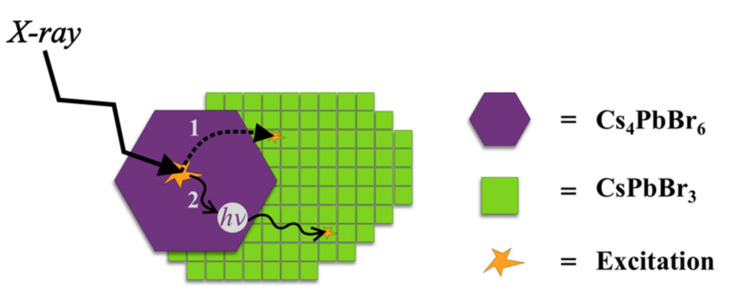
Schematic illustration of the energy-transfer hypothesis; **path 1**: non-radiative energy transfer from Cs_4_PbBr_6_ excited state to the excited state of neighboring CsPbBr_3_ nanocrystal; **path 2**: radiative energy transfer, ultraviolet photon emitted by scintillation process in Cs_4_PbBr_6_ is absorbed by CsPbBr_3_ nanocrystal.

Cs_4_PbBr_6_ has larger band gap than CsPbBr_3_, therefore an energy transfer is theoretically possible. The drop in the mixed sample excitation spectra does not go all the way to zero intensity, so it does not rule out this possibility as well. We tested the following hypothesis (see Figure 4): Is it possible that the incident radiation excites the Cs_4_PbBr_6_ phase, and then the energy is either radiatively or non-radiatively transferred to the CsPbBr_3_ phase? TEM shows that both phases are in a very close proximity, so both mechanisms are, in principle, possible, even if the radiative transfer has in this case generally much higher probability.

In order to investigate the possible energy transfer between these two phases, we recorded PL decays at two excitation wavelengths for both the pure and the mixed sample. One wavelength (310 nm) was selected to excite mostly the Cs_4_PbBr_6_ phase, and the second (389 nm) to excite the CsPbBr_3_ phase exclusively.

Decay curves of the pure sample are shown in Figure 3c,d. They are almost identical, featuring 6 ns fast decay component. Panels (e) and (f) show decay curves of the mixed sample. Again, they are almost identical, therefore no energy transfer from Cs_4_PbBr_6_ to CsPbBr_3_ was confirmed. Moreover, compared to the pure sample, the fast components are roughly similar, only the slowest component seems to be faster in the mixed sample. Additionally, the contribution of the fastest component is significantly higher in the mixed sample. 

This acceleration of the decay time in the mixed sample is probably caused by the presence of smaller CsPbBr_3_ nanocrystals. One factor may be the quantum confinement effect, but it can also be caused by the luminescence quenching on various defects. It is well known that, in smaller nanocrystals with higher surface to volume ratio, more surface defects are present, which can be responsible for significant quenching. Nevertheless, the presence of Cs_4_PbBr_6_ phase seems to have no effect on PL temporal characteristics of the CsPbBr_3_ nanocrystals, which might also be due to severe thermal quenching of the emission of the former at room temperature [34].

**Figure 5:**
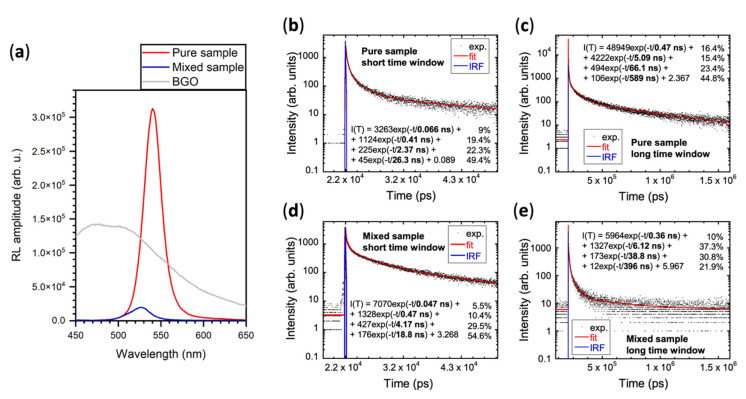
(**a**) Radioluminescence (RL) spectra of the pure sample (red line) and of the mixed sample (blue line) compared to the RL spectrum of Bi_4_Ge_3_O_12_ (BGO) powder (grey line); (**b**–**e**) scintillation decay curves for both the pure (**b**,**c**) and mixed (**d**,**e**) samples, recorded in both the short (**b**,**d**) and long (**c**,**e**) time windows. Black dots represent the experimental data, red line is the best fit (4-exponential function), and blue line is the instrumental response function (IRF).

Due to the nature of the samples, it is challenging to guarantee the same concentration of the solid phase in both mixed and pure samples to reliably assess the quantitative effect of the Cs_4_PbBr_6_ presence on the CsPbBr_3_ PL intensity. This is also the reason we present only normalized (to a maximum) PL spectra in Figure 3b. However, we can ensure the same thickness of the centrifuged solid samples for radioluminescence (RL) characterization, and thus provide the quantitative comparison in this set of data (cf. Figure 5). Moreover, as the target application of our investigation is the high energy radiation detection, quantitative changes in scintillation (unlike PL) parameters are those of real interest.

Figure 5 summarizes RL characterization of both samples with powder Bi_3_Ge_4_O_12_ (BGO) standard scintillator used for a comparison. Steady state RL spectrum in panel (a) shows that intensity of the pure sample is one order of magnitude larger than that of the mixed sample. One factor contributing to such difference may be the abovementioned higher concentration of surface defects resulting from the smaller CsPbBr_3_ nanocrystals present in the mixed sample. However, this alone would not cause such a strong effect. Furthermore, it can be expected that CsPbBr_3_ nanocrystals prepared at room temperature by rapid precipitation process would have poorer crystallinity, more crystallographic defects, and, subsequently, lower PLQY, compared to nanocrystals precipitated at elevated temperatures during the hot injection process. However, we have never encountered any evidence in the literature about CsPbBr_3_ nanocrystals prepared by the precipitation method and compared to CsPbBr_3_ nanocrystals prepared by the hot injection in the same lab to have such poor photoluminescence properties that could result in one order of magnitude difference in scintillation light output.

On the other hand, the presence of Cs_4_PbBr_6_ crystals in the sample is capable of significantly deteriorating the bright luminescence of CsPbBr_3_ nanocrystals due to the emission dumping effect at Cs_4_PbBr_6_ caused by its strong quenching [34]. Figure 3b shows a significant drop in the excitation spectrum of CsPbBr_3_ emission resulting from the Cs_4_PbBr_6_ absorption. Neither PL decay measurements in Figure 3, nor scintillation decay measurements in Figure 5, indicate any form of energy transfer from Cs_4_PbBr_6_ to the CsPbBr_3_ phase (due to thermal quenching of its emission). Therefore, all the energy that is deposited in Cs_4_PbBr_6_ crystals is lost to the scintillation process in CsPbBr_3_. Moreover, Cs_4_PbBr_6_ crystals are one order of magnitude larger than CsPbBr_3_ nanocrystals in the mixed sample, therefore they are more capable of efficient stopping the incident X-ray radiation. In addition, they are diluting the CsPbBr_3_ concentration in this sample, which further reduces the probability of effective deposition of incident radiation energy in the CsPbBr_3_ phase.

Furthermore, incident radiation generates excitons, or self-trapped electrons and holes, in the Cs_4_PbBr_6_ lattice. When localized charge carriers diffusing through Cs_4_PbBr_6_ encounter large and fairly even offset at the conduction and valence band edges (Cs_4_PbBr_6_ vs. CsPbBr_3_), they will likely dissociate, and it may serve to concentrate carriers in CsPbBr_3_ [46]. Smaller Cs_4_PbBr_6_ particles would trigger shorter diffusion length and consequently higher probability of dissociation and charge transfer to CsPbBr_3_, again resulting in better efficiency and yield of green emission.

The larger the Cs_4_PbBr_6_ crystals, the more prominent the above-described effects reducing the overall RL intensity.

Therefore, we conclude that the presence of Cs_4_PbBr_6_ crystals alongside CsPbBr_3_ nanocrystals significantly reduces their scintillation light output.

This conclusion supports theoretical prediction published in the recent Perspective [45]. They considered a CsPbBr_3_@Cs_4_PbBr_6_ quantum-dot-in-host-like composite, and calculated a PL spectrum escaping from 10 μm depth within the sample. They found that, compared to the launched PL spectrum, the escaping one is 100× attenuated and red-shifted by 20 nm. Our experiments qualitatively confirm this weakening of CsPbBr_3_ light output in the presence of Cs_4_PbBr_6_ phase. Our systems were not identical, but in both cases, it was the CsPbBr_3_ nanocrystalline phase surrounded by a larger amount of Cs_4_PbBr_6_ phase in some form, therefore we believe that this comparison is justified. We also confirm a significant red shift (15 nm) between the PL spectrum of colloidal sample (i.e., “launched” spectrum) and RL spectrum of precipitated powder (i.e., “escaping” spectrum from within the sample). This red shift also occurs in the pure sample, where it is even larger (23 nm) due to the higher concentration of absorbing CsPbBr_3_ nanocrystals (Cs_4_PbBr_6_ phase does not absorb the 517 nm light).

Scintillation decay curves of both samples are similar within the uncertainty given by the 4-exponential approximation. They all feature two sub-nanosecond components (50 ps and 400 ps), which is a crucial property for the intended fast timing applications.

The presence of Cs_4_PbBr_6_ phase does not affect luminescence properties of the sample, other than the scintillation light output. Therefore, for most optoelectronic applications, its presence does not hinder successful implementation. It can even prove beneficial in protecting CsPbBr_3_ nanocrystals from a deteriorative effect of air oxygen and humidity, as in [23,24,25]. However, for applications such as scintillation detectors for fast timing, the drop in the CsPbBr_3_ radioluminescence intensity can become detrimental.

## 4. Conclusions

We synthesized and characterized CsPbBr_3_ nanocrystals prepared by the hot injection method (HI) and their mixture with Cs_4_PbBr_6_ crystals using the room temperature precipitation method (RTP), which we compared and evaluated with respect to possible future optoelectronic applications. Our RTP protocol yields high amount of Cs_4_PbBr_6_ phase, which allowed us to study its possible effect on the CsPbBr_3_ luminescence properties.

We demonstrated that the Cs_4_PbBr_6_ crystals have significant negative impact on the CsPbBr_3_ scintillation light output, most probably due to strong thermal quenching of their luminescence, but do not affect timing properties in any way. This conclusion supports theoretical predictions in [45] even if our system was not identical. We believe that this is another step towards better understanding of such materials regarding their scintillation characteristics. Moreover, our study did not provide any sufficient evidence of an energy transfer between those two phases.

We conclude that the presence of Cs_4_PbBr_6_ phase should be a concern for any optoelectronic application requiring high scintillation light output, such as scintillation detectors for fast timing applications. In this case, much attention needs to be paid to characterization of the material prepared by the RTP process to rule out the possible presence of Cs_4_PbBr_6_ phase, especially when thinking of upscaling for large batches for possible future industrial production.

## Data Availability

The data presented in this study are available on request from the corresponding author.

## Supplementary Materials

The following are available online at https://www.mdpi.com/article/10.3390/nano11081935/s1, Figure S1: Structure of CsPbBr_3_ and Cs_4_PbBr_6_ drawn with VESTA software [S1], details on syntheses, Figure S2: Absorption spectra of the pure sample dispersed in hexane in three different concentrations; absorption spectra of the surfactants in the inset, Figure S3: Emission spectrum (excited at 300 nm) of the mixed sample and corresponding excitation spectra for each emission band, Figure S4: Gaussian decomposition of the mixed sample emission spectrum, and Figure S5: Comparison of PL of both samples excited by the 365 nm UV light. Reference [56] is cited in the supplementary materials.
