# Supplementary material for: On the Role of Cs4PbBr6 Phase in the Luminescence Performance of Bright CsPbBr3 Nanocrystals
Source: Nanomaterials (Basel). 2021 Jul 27;11(8):1935. doi: 10.3390/nano11081935 (PMC8400622; doi:10.3390/nano11081935)
Supplement: Supplementary file 1 [file nanomaterials-11-01935-s001.zip › nanomaterials-1305410-supplementary.pdf]

Supplementary Materials

On the Role of Cs_4PbBr_6 Phase in the Luminescence Performance of Bright $CsPbBr_3$ Nanocrystals

Kateřina Děcká [1,2,*], Adéla Suchá [1], Jan Král [1], Ivo Jakubec [3], Martin Nikl [2], Vítězslav Jarý [2], Vladimir Babin [2],
Eva Mihóková [2] and Václav Čuba [1]

[1] Department of Nuclear Chemistry, Faculty of Nuclear Sciences and Physical Engineering, Czech Technical University in Prague, Břehová 7, 115 19 Prague 1, Czech Republic; suchaade@fjfi.cvut.cz (A.S.); kralja13@fjfi.cvut.cz (J.K.); vaclav.cuba@fjfi.cvut.cz (V.Č.)
[2] Institute of Physics, Czech Academy of Sciences, Cukrovarnická 10, 162 00 Prague 6, Czech Republic; nikl@fzu.cz (M.N.); jary@fzu.cz (V.J.); babinv@fzu.cz (V.B.); mihokova@fzu.cz (E.M.)
[3] Institute of Inorganic Chemistry, Czech Academy of Sciences, Husinec-Řež č.p. 1001, 250 68 Řež, Czech Republic; jakubec@iic.cas.cz
* Correspondence: Katerina.Decka@fjfi.cvut.cz

Structure

Figure S1 shows the structure of orthorhombic $CsPbBr_3$ and rhombohedral Cs_4PbBr_6. Note that the blue octahedrons $(PbBr_6)^-$ are interconnected in the case of $CsPbBr_3$ (left), but isolated in the case of Cs_4PbBr_6 (right).

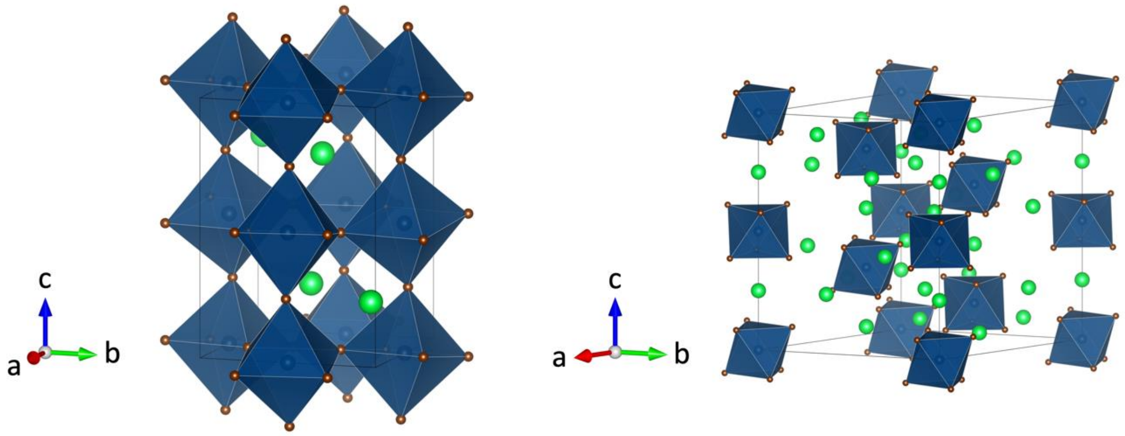

Figure S1. Structure of $CsPbBr_3$ (**left**) and Cs_4PbBr_6 (**right**) drawn with VESTA software [1]. Green atoms are Cs, blue atoms are Pb, and brown atoms are Br. For better clarity and readability, the Cs–Br bonds were omitted in this drawing and the atom sizes were reduced to a half.

Details on syntheses

Cs-oleate (0.4M) Synthesis

A total of 1.303 g of cesium carbonate (4 mmol), 12.7 mL of oleic acid (40 mmol), and 7.3 mL of 1-octadecene was loaded into 100mL three-necked flask and connected to a Schlenk line. The reaction mixture was subjected to two cycles of evacuation under vigorous stirring and refilling with argon, and then was heated up under vacuum to 110 °C. After 1 h, all cesium carbonate was dissolved, and the solution was left to cool down to room temperature. The synthesized cesium oleate stock solution was stored under inert atmosphere and was dried for 1 h under vacuum at 110 °C before each use.

$CsPbBr_3$ Synthesis by HI Method

A total of 0.276 g of $PbBr_2$ (0.752 mmol), 20 mL of octadecane, 2 mL of oleylamine, and 1.78 mL of oleic acid were loaded into 100ml three-necked flask and connected to a Schlenk line. The reaction mixture was subjected to two cycles of evacuation under vigorous stirring and refilling with argon, and then was heated up under vacuum to 110 °C. After 1 h, all $PbBr_2$ was dissolved, and temperature was raised under argon atmosphere to 170 °C. The injection of 0.5 ml of dried 0.4 M cesium oleate solution was performed, and after 10 s the reaction mixture was cooled down in an ice-water bath. The synthesized nanocrystals were isolated by centrifugation at 10,950 × g for 5 min and redispersed in hexane. The sample for the X-ray diffraction and radioluminescence characterization was obtained after the first centrifugation step before redispersion. The second centrifugation under the same conditions was performed to improve the size uniformity of the $CsPbBr_3$ nanocrystals. Sedimented particles were discarded, and supernatant was characterized (TEM, absorption spectra, photoluminescence emission, and excitation spectra).

$CsPbBr_3$-Cs_4PbBr_6 Synthesis by RTP Method

In a typical synthesis of $CsPbBr_3$-Cs_4PbBr_6 mixture by RTP method, 0.1468 g of $PbBr_2$ (0.4 mmol) and 0.0851 g of CsBr (0.4 mmol) were loaded in 10 ml of DMF. After 1 h of stirring, 0.5 ml of oleylamine and 1 ml of oleic acid were added to the solution. Then, 1 ml of the solution was quickly added into vigorously stirred toluene (10 mL) which caused rapid precipitation. The reaction was left to proceed for 1 min. Light yellow suspension with strong green PL emission under UV (365 nm) light irradiation was obtained. Then, the solution was centrifuged at (4088 × g) for 1 min. The precipitate was redispersed in toluene and characterized (TEM, absorption spectra, photoluminescence emission, and excitation spectra). Supernatant was also characterized (absorption spectra, photoluminescence emission, and excitation spectra). A sample for X-ray diffraction and radioluminescence characterization was obtained after the centrifugation step before redispersion. The supernatant sample for X-ray diffraction was prepared by drop-casting on a sample holder mildly heated by a hot plate. Each drop was left to evaporate, the casting proceeded until a visible film was obtained. All the experimental procedures were operated at room temperature and free from inert gas.

Supplementary Data

Absorption of surfactants

Figure S2 shows absorption spectra of the pure sample at three different concentrations. Note that the relative intensity of the absorption band peaking at 261 nm decreases with the decreasing $CsPbBr_3$ concentration. This suggests that this absorption band is directly connected to the overall concentration of $CsPbBr_3$ nanocrystals and is not caused by the solvent. We conclude that this peak is caused by an excess of oleic acid and oleylamine in the sample, which is confirmed by their absorption spectra (see the inset).

Emission and Excitation Spectra of Supernatant

Figure S3 shows photoluminescence spectra of the supernatant of the mixed sample. The emission spectrum features two bands (see their Gaussian decomposition in Figure S4) in the blue region, significantly blue-shifted from the spectrum of the precipitate presented in Figure 3 in the main article. This suggests that only the smallest $CsPbBr_3$ nanocrystals remained in the supernatant after the centrifugation. The two emission bands probably correspond to different sizes of the nanocrystals, or rather, to different thicknesses of the nanoplatelets. The presence of the nanoplatelets in this sample is argued in the main article, supported by XRPD and absorption data. The corresponding excitation spectra for the two peaks are also presented in Figure S3.

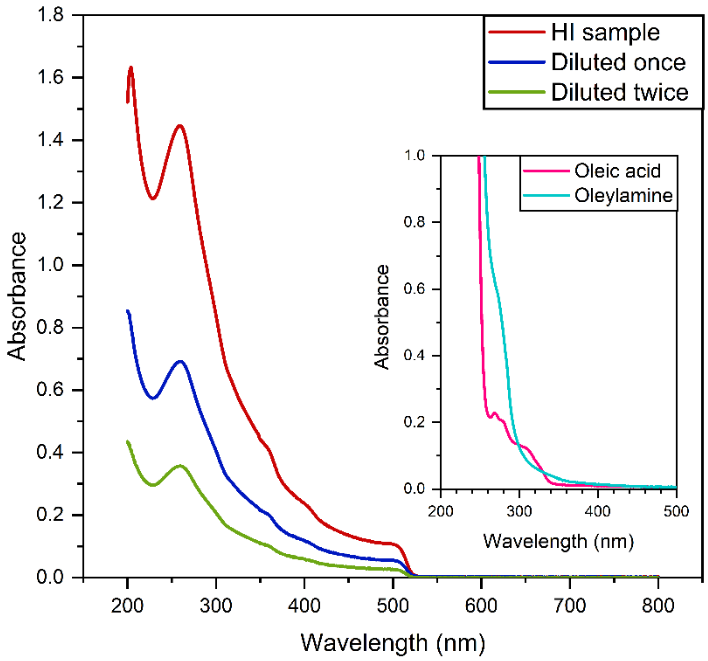

Figure S2. Absorption spectra of the pure sample dispersed in hexane in three different concentrations: no dilution (red line), once diluted (blue line, half of the original concentration), and twice diluted (green line, quarter of the original concentration). The graph in the inset shows the absorption spectra of the surfactants used in the synthesis, namely oleic acid (red line), and oleylamine (blue line).

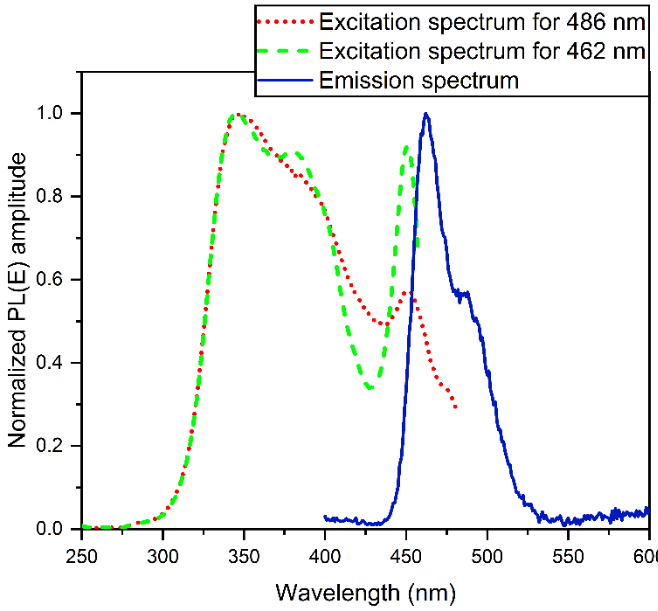

Figure S3. Emission spectrum (excited at 300 nm) of the mixed sample (blue solid line) and corresponding excitation spectra. Green dashed line is an excitation spectrum for the 462 nm emission band and the red dotted line is an excitation spectrum for the 486 nm emission band.

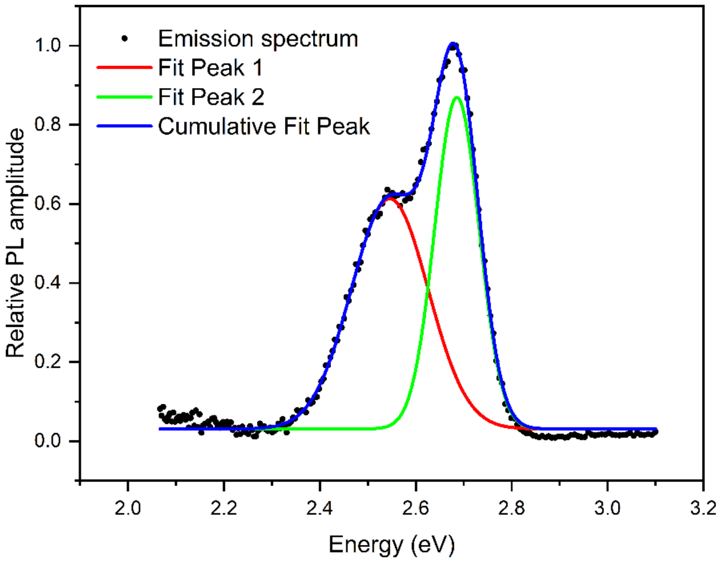

Model	Gauss	
Equation	y=y0 + (A/(w*sqrt(pi/2)))*exp(-2*((x-xc)/w)^2)	
Plot	Peak1(Relative PL amplitude)	Peak2(Relative PL amplitude)
y0	0.031 ± 0.00166	0.031 ± 0.00166
xc	2.54561 ± 0.00222	2.68572 ± 7.42245E-4
w	0.16032 ± 0.00344	0.09267 ± 0.00128
A	0.11696 ± 0.00294	0.09739 ± 0.00267
Reduced Chi-Sqr	3.05688E-4	
R-Square (COD)	0.99653	
Adj. R-Square	0.99642	

Figure S4. Gaussian decomposition of the mixed sample emission spectrum. Black dots are experimental data, blue line is the double Gaussian fit, and red and green lines are Gaussian components of the emission spectrum, respectively. The table summarizes details of the fit parameters.

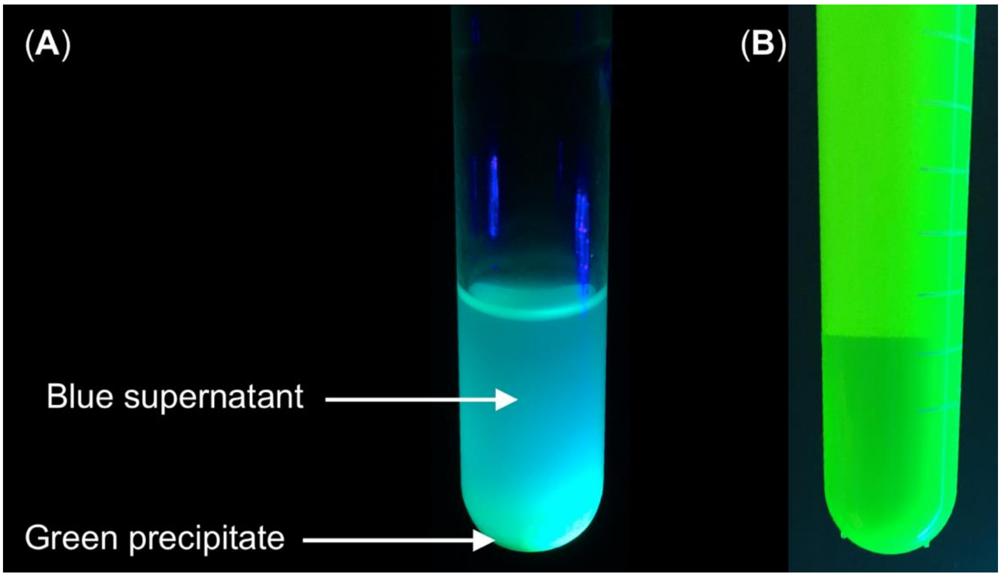

Figure S5. Comparison of PL of both samples excited by the 365 nm UV light. (**A**) mixed sample; (**B**) pure sample.

References

1. Momma, K.; Izumi, F. VESTA3 for three-dimensional visualization of crystal, volumetric and morphology data. *J. Appl. Crystallogr*. **2011**, *44*, 1272–1276, doi:10.1107/S0021889811038970.
